# Limb-Girdle Muscular Dystrophies (LGMDs): The Clinical Application of NGS Analysis, a Family Case Report

**DOI:** 10.3389/fneur.2019.00619

**Published:** 2019-06-13

**Authors:** Claudia Strafella, Giulia Campoli, Rosaria Maria Galota, Valerio Caputo, Giulia Pagliaroli, Stefania Carboni, Stefania Zampatti, Cristina Peconi, Julia Mela, Cristina Sancricca, Guido Primiano, Giulietta Minozzi, Serenella Servidei, Raffaella Cascella, Emiliano Giardina

**Affiliations:** ^1^Molecular Genetics Laboratory Unione Italiana Lotta Distrofia Muscolare (UILDM), Santa Lucia Foundation, Rome, Italy; ^2^Department of Biomedicine and Prevention, Tor Vergata University, Rome, Italy; ^3^Fondazione Policlinico Universitario A. Gemelli IRCCS, UOC Neurofisiopatologia, Rome, Italy; ^4^Unione Italiana Lotta Distrofia Muscolare (UILDM), Sezione Laziale, Rome, Italy; ^5^Department of Veterinary Medicine, University of Milan, Milan, Italy; ^6^Department of Biomedical Sciences, Catholic University Our Lady of Good Counsel, Tirana, Albania

**Keywords:** LGMDs, *CAPN3*, *LMNA*, NGS panel, familial investigation, calpainopathy, cardiovascular disease

## Abstract

The diagnosis of LGMD2A (calpainopathy) can be challenging due to genetic heterogeneity and to high similarity with other LGMDs or neuromuscular disorders. In this setting, NGS panels are highly recommended to perform differential diagnosis, identify new causative mutations and enable genotype-phenotype correlations. In this manuscript, the case of a patient affected by LGMD2A is reported, for which the application of a defined custom designed NGS panel allowed to confirm the diagnosis of calpainopathy linked with two heterozygous variants in *CAPN3*, namely c.550delA and c.1813G>C. The first variant has been extensively described in relation to calpainopathy. The second variant c.1813G>C, instead, is novel and has been predicted to be probably damaging. In addition, NGS analysis on the proband revealed a heterozygous variant (c.550C>T) in the *LMNA* gene, which is associated with dilated cardiomyopathy. The variant is novel and has been predicted to be deleterious by subsequent bioinformatic analysis. Successively, segregation analysis was performed on family members. Interestingly, none of them showed neuromuscular symptoms but the mother was diagnosed with bradycardia and syncopal episodes and showed a positive family history for cardiomyopathy. The segregation analysis reported that the proband inherited the c.1813G>C (*CAPN3*) from the father who was a healthy carrier. The mother was positive for c.550delA (*CAPN3*) and c.550C>T (*LMNA*), suggesting thereby a possible genetic explanation for her cardiovascular problems. Segregation analysis, therefore, confirmed the inheritance pattern of the variants carried by the proband and highlighted a familiarity for cardiomyopathy which should not be neglected. The NGS analysis was further performed on the partner of the proband, to estimate the reproductive risk of the couple. The partner was negative to NGS screening, suggesting thereby a low risk to have an affected child with calpainopathy and 50% probability to inherit the *LMNA* variant. This case report showed the clinical utility of the NGS panel in providing accurate LGMD2A diagnosis and identifying complex phenotypes originating from mutations in multiple genes. However, NGS results should always be accomplished by a dedicated genetic counseling, not only to evaluate the recurrence and reproductive risks but also to uncover unexpected findings which can be clinically significant.

## Introduction

The Limb-Girdle Muscular Dystrophies (LGMDs) include a heterogenous group of disorders characterized by the progressive wasting and weakness of the proximal limb-girdles muscles (1, 2). LGMDs display inter and intrafamilial variability, ranging from very mild forms, to severe, early onset, rapidly progressive phenotypes (2). LGMDs can be classified as autosomal dominant (LGMD1) or recessive (LGMD2). The first group usually has an adult age of onset and are not very common (<10% of all LGMDs), whereas the latter are more frequent (1:15000) (1). Among recessive forms, the LGMD2A (or calpainopathy) is the most common LGMD worldwide, affecting approximately 30% of all LGMDs cases (3). Clinical signatures of disease include tiptoe walking, waddling gait, difficulty in running and climbing stairs, scapular winging. Joint contractures, Achilles tendon shortening, scoliosis are commonly observed, while facial and neck muscles are not affected (4). An asymptomatic HyperCKemia (5–80 times CPK normal levels) in young patients is considered a preclinical stage of disease and may persist for several years (1, 5). The age of onset of muscle weakness usually occurs at 15 years, although it may arise at earlier (<12 years) or later (>30 years) ages. The disease progression can lead to loss of ambulation, respiratory insufficiency, and reduced lung vital capacity in the advanced stages (6). Cardiac involvement (cardiac rhythm disorders, cardiac conduction disorders, left ventricular ejection dysfunction) is only occasionally reported (6, 7).

The diagnosis of calpainopathy is confirmed by the detection of pathogenic mutations in *CAPN3* (15q15.1) (4), encoding different alternatively spliced transcripts. However, the full-length transcript is primarily expressed in muscle tissue (8). The encoded protein (CAPN3) is a member of non-lysosomal Ca^++^-dependent cysteine proteases family. In muscle, CAPN3 takes part in “sarcomere remodeling,” which is essential for muscle adaption and growth in response to functional and metabolic demands. To date, more than 490 pathogenic mutations throughout *CAPN3* have been described, most of which are single-nucleotide changes (4). Mutations in *CAPN3* have been associated with mitochondrial abnormalities, growth failure, increased oxidative stress and sarcomere disorganization which altogether contribute to make the muscle unable to hold loads, causing thereby myofiber degeneration and muscle wasting (8). The diagnosis of calpainopathy can be challenging because of the genetic heterogeneity and the non-specificity of clinical and instrumental pattern. Indeed, distribution of muscular weakness/atrophy, hypertrophy/pseudo-hypertrophy, and tendon contractures are very often shared with other LGMDs or neuromuscular disorders. Muscle biopsy pattern in calpainopathy is generally non-specific too, ranging from mild muscular abnormalities to severe dystrophic changes. Moreover, immunohistochemical/biochemical markers are usually not reliable: calpain signal can be normal even in presence of a non-functional protein, and vice versa it can be reduced even in other muscular dystrophies different from calpainopathy.

It is therefore recommended to perform a differential diagnosis in order to provide accurate and reliable results. To this purpose, molecular genetic testing approaches have been developed to confirm the diagnosis of calpainopathy, including multigene panel, extensive genomic (exome/genome sequencing) and single-gene analysis (direct sequencing) (9). The implementation of Next-Generation Sequencing (NGS) was helpful to generate informative data to be applied to diagnostic, predictive or therapeutic purposes (10–12). NGS gene panels are based on the analysis of a set of genes associated with a specific disease or a group of related disorders, which are characterized by genetic and phenotypic heterogeneity. NGS panels can also be useful for detecting blended or complex phenotypes, which result from the inheritance of more than one genetic defect from parents (13). In general, patients presenting complex phenotypes, who are negative to the known mutations in custom-designed diagnostic gene panels and requiring a broad differential diagnosis, are eligible for whole exome or genome sequencing. Whole exome/genome sequencing are more expensive approaches in terms of data management, interpretation and analytical costs but increase the likelihood of providing a molecular diagnosis to a suspected genetic disorder (13). Concerning LGMDs, dedicated NGS panels are highly recommended, to ensure high diagnostic rates, optimal coverage, sensitivity and specificity of clinical testing (9). NGS panels represent therefore one of the best systems to facilitate differential diagnosis, identify new causative mutations and clarify genotype-phenotype correlations (14, 15).

In this manuscript, the case of a patient affected by LGMD2A is reported, for which the application of the NGS panel allowed not only to confirm the diagnosis of calpainopathy (mutations in *CAPN3*) but also to identify an additional, novel mutation in *LMNA* gene associated with dilated cardiomyopathy. Given these results, the analysis was extended to the family members of the proband to provide a more comprehensive interpretation of the analytical data in relation to the pathological phenotypes.

## Case Presentation

### Clinical Characterization of Proband and Relatives

**Figure 1:**
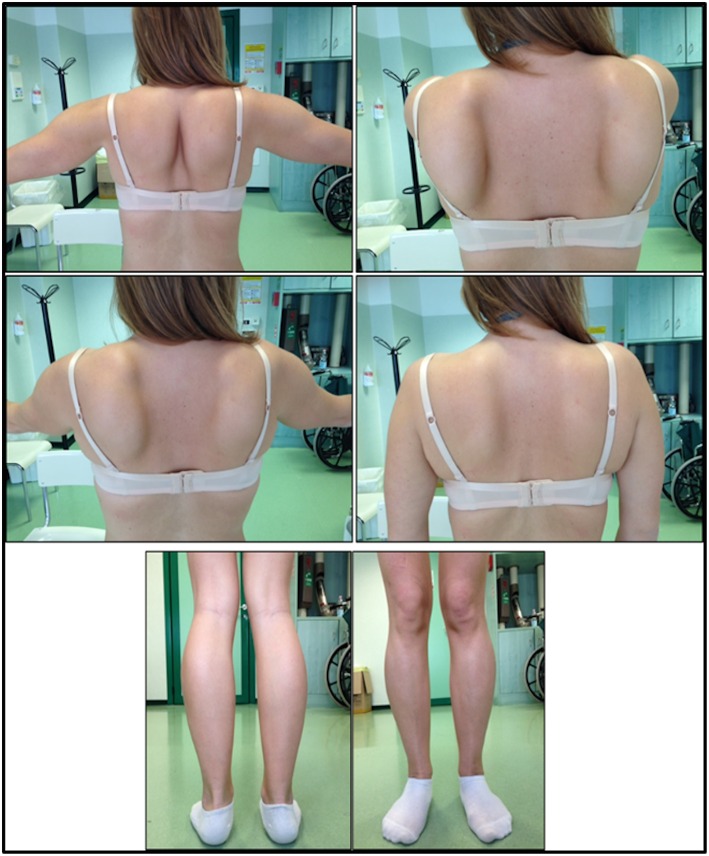
Proband clinical features: winged scapulae and combination of thigh atrophy and calf pseudo-hypertrophy.

**Figure 2:**
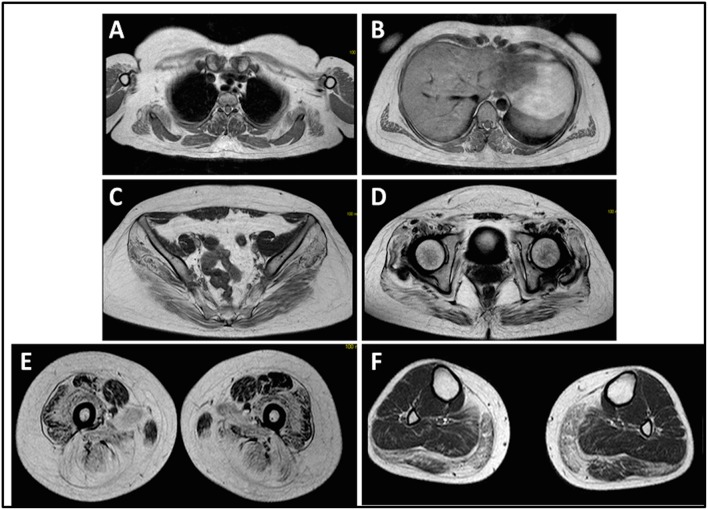
Proband muscle MRI: involvement of upper limb girdle and paravertebral muscles **(A,B)**, involvement of lower limb girdle and posterior compartment of the thigh (**C–E**, mostly the glutei) with relative sparing of the sartorius and gracilis, and involvement of the posterior compartment of the leg (**F**, mostly gastrocnemius medialis/soleus).

The onset of disease in the proband was referred at 10 years of age, when she reports the presence of calf hypertrophy with tiptoe walking, difficulties in running, climbing stairs, and standing-up. Symptoms showed a slow but progressive worsening, with subsequent involvement of proximal upper limb. At the age of 13, high levels of CPK (~8,000 UI/L) were incidentally discovered, never associated with myoglobinuria. Successively, patient underwent several neuromuscular investigations at various specialized centers. Two muscular biopsies were performed, showing classic dystrophic picture (hypertrophic/atrophic fibers, internal nuclei, necrotic fibers, increase in connective tissue) with non-specific characteristics. As expected, immunochemistry and immunoblot studies were inconclusive indicating abnormal/reduced α, β, γ sarcoglycan, β-dystroglycan, and dystrophin signal only in necrotic fibers (repeated immunochemistry resulted normal, also for laminin); immunoblots for dystrophin and calpain revealed normal signals. However, the calpain-3 immunoblot is known to be not-completely sensitive for LGMD2A diagnosis, since 20–30% of cases display a normal quantity of protein (1, 16–18). Due to the clinical presentation (hyperCKmia, calf hypertrophy/pseudo-hypertrophy) dystrophinopathies were firstly ruled out. In addition, analysis of *DMD* (Xp21), *FKRP* (19q13.32), and *DYSF* (2p13.2) genes did not reveal pathogenic mutations. Patient came to our observation at the age of 30, presenting axial and girdle involvement both in upper and lower limb, with significant waddling gait and winged scapulae. In lower limbs, weakness was prominent in quadriceps and glutei muscles which were significantly ipo-atrophic, together with the evidence of “apparently hypertrophic” (pseudo-hypertrophy) calf muscles (Figure 1). Cramps, myalgias, and rippling were not observed. Complete pneumological (spirometry and polysomnography) and cardiological (echography, 24-h ECG Holter monitoring, cardiac MRI, complete cardiological physical inspection with targeted medical history and cardiovascular reflex analysis) examinations did not reveal any significant abnormality. We also ruled out acid maltase deficiency (normal enzyme activity assays in lymphocytes/leukocytes). Muscle Magnetic Resonance Imaging (MRI) showed a prominent involvement of scapular girdle, paravertebral muscles, posterior compartment muscles of the thigh (with relative sparing of the sartorius and gracilis muscles) and of the leg (in particular gastrocnemius medialis and soleus) (Figure 2). This pattern of involvement has already been reported in literature concerning MRI picture in calpainopathy (19, 20). In addition, the MRI study showed that calf muscles were effectively atrophic and characterized by fat infiltration, causing a muscular “enlargement.” The clinical presentation, the age of onset, the rate of progression, the distribution of muscle weakness and the MRI findings in the proband were fully consistent with a diagnosis of LGMD2A extensively described in literature (4, 21, 22).

The evaluation of the family members of the proband did not revealed any history of neuromuscular disease and no evidence of consanguinity between parents. However, the mother of the proband, at the age of 55, presented 2 syncopal episodes for which she was diagnosed with idiopathic bradycardia (up to 40 bpm by night). Extensive cardiological investigations on the mother of the proband diagnosed a symptomatic atrioventricular (AV) block after the occurrence of several syncopal episodes/lipothymia. The analysis showed the presence of a basic first-degree AV block, with several periods of severe bradycardia, mostly nocturnal and often symptomatic, associated with some phases of second-degree AV block, both type 1 and 2, and some phases of nocturnal complete AV dissociation. Echocardiography and ergometric test did not reveal significant alterations, excepted for the patent foramen ovale. The mother did not present any other clinical symptom, predisposing condition or risk factor. Given her clinical picture, the mother of the proband was implanted with PMK. In addition, family history revealed that the grandmother and the great-grandfather of the proband were also implanted with PMK at the age of 55 and 30 years, respectively. Moreover, the brother of the grandmother of the proband died at 51 for severe dilated cardiomyopathy. Cardiological examinations were also performed on the father and the maternal uncle of the proband who resulted completely unaffected.

The present study was approved by the ethics committee of Santa Lucia Foundation and was performed according to the Declaration of Helsinki. All participants provided signed informed consent for genetic analysis, and, in this regard, they also provided the consent for publication of this case report.

### Laboratory Investigations and Diagnostic Tests

Genomic DNA was extracted from peripheral blood (400 μL) using MagPurix Blood DNA Extraction Kit and MagPurix Automatic Extraction System (Resnova) according to the manufacturer's instructions. Samples were sequenced using Ion PGM System and Ion Ampliseq Customized Panel High Specificity (Thermo Fisher Scientific). The size of the panel was 129.13 Kb, which is expected to screen ~99.72% of the total panel with a minimum coverage of 20X. The panel included 18 genes, which were selected using scientific literature, GeneReviews (www.ncbi.nlm.nih.gov/books/NBK1116/) and frequency of pathogenic variants in the general population. A detailed description of the NGS panel has been summarized in Table 1.

**Table 1:** Customized NGS panel utilized for LGMD diagnosis.

**Gene**	**OMIM number**	**Locus**	**Phenotype (Inheritance)**	**Size of the target region (bp)**	**Exon**	**Transcript ID**	**Coverage (%)**
*DES*	125660	2q35	Muscular dystrophy, limb-girdle, type 2R (AR)	2,338	9	ENST00000373960.3	100
*DNAJB6*	611332	7q36.3	Muscular dystrophy, limb-girdle, type 1E (AD)	3,290	10	ENST00000262177.8	99,91
*EMD*	300384	Xq28	Emery-Dreifuss muscular dystrophy 1 (XLR)	1,399	6	ENST00000369842.8	100
*MYOT*	604103	5q31.2	Myopathy, myofibrillar, 3 or Muscular dystrophy, limb-girdle, type 1A (AD)	2,372	10	ENST00000239926.8	100
*LMNA*	150330	1q22	Emery-Dreifuss muscular dystrophy 2 or Muscular dystrophy, limb-girdle, type 1B (AD)	4,274	12	ENST00000368300.8	99,44
*CAV3*	601253	3p25.3	Muscular dystrophy, limb-girdle, type 1C (AD, AR)	1,451	2	ENST00000343849.2	100
*TNPO3*	610032	7q32.1.	Muscular dystrophy, limb-girdle, type 1F (AD)	4,813	23	ENST00000265388.9	100
*CAPN3*	114240	15q15.1	Muscular dystrophy, limb-girdle, type 2A (AR)	3,922	24	ENST00000397163.7	100
*DYSF*	603009	2p13.2	Muscular dystrophy, limb-girdle, type 2B (AR)	7,858	55	ENST00000258104.7	100
*SGCG*	608896	13q12.12	Muscular dystrophy, limb-girdle, type 2C (AR)	1,735	8	ENST00000218867.3	100
*SGCA*	600119	17q21.33	Muscular dystrophy, limb-girdle, type 2D (AR)	1,533	10	ENST00000262018.7	100
*SGCB*	600900	4q12	Muscular dystrophy, limb-girdle, type 2E (AR)	4,339	6	ENST00000381431.9	98,62
*SGCD*	601411	5q33.2-q33.3	Muscular dystrophy, limb-girdle, type 2F (AR)	10,234	9	ENST00000337851.8	100
*TCAP*	604488	17q12	Muscular dystrophy, limb-girdle, type 2G (AR)	983	2	ENST00000309889.2	100
*FKTN*	607440	9q31.2	Muscular dystrophy-dystroglycanopathy (limb-girdle), type C, 4 (AR)	7,566	11	ENST00000602661.5	99,68
*ANO5*	608662	11p14.3	Muscular dystrophy, limb-girdle, type 2L (AR)	6,881	22	ENST00000324559.8	98,75
*FKRP*	606596	19q13.32	Muscular dystrophy-dystroglycanopathy (limb-girdle), type C, 5 (AR)	3,514	4	ENST00000318584.9	99,37
*SMCHD1*	614982	18p11.32	Facioscapulohumeral muscular dystrophy 2, digenic (AD)	9,152	48	ENST00000320876.10	100

*AD, Autosomal Dominant; AR, Autosomal Recessive; XLR, X-linked Recessive*.

Libraries construction was performed by Ion AmpliSeq™ Library Kits 2.0. Approximately 10 ng/μl of starting DNA were utilized for multiplex PCR reactions. Successively, two purification steps (using AMPure XP, Beckman Coulter) were performed to remove unwanted contaminants and a final PCR was performed. Template amplification and enrichment steps were performed by Ion PGM Hi-Q OT2 kit-400, Ion OneTouch 2 System and Ion OneTouch ES (Thermo Fisher Scientific). Samples were processed by Ion PGM Hi-Q Sequencing Kit (400 bp, Thermo Fisher Scientific) and run on Ion 316 Chip v2 (850 flows required) and Ion PGM Sequencer (Thermo Fisher Scientific). The results were analyzed using Ion Reporter 4.6 (Thermo Fisher Scientific) and Integrated Genome Viewer (IGV). The interpretation of genetic variants was conducted by Human Gene Mutation Database (HGMD), Leiden Open Variation Database (LOVD), ClinVar and ExAC. The functional effect of the detected variants was evaluated by bioinformatic predictive tools, including Mutation Taster, Varsome, SIFT, PolyPhen 2, SMART, Human Splicing Finder (HSF). Direct sequencing (BigDye Terminator v3.1, BigDyeX Terminator and ABI3130, Applied Biosystems) was performed to confirm genetic variants and to sequence genomic coding regions with a coverage <20X (*LMNA*, Chr1:156106052-156106076; *DYSF*, Chr2:71753352-71753502, Chr2:71776385-71776640; *SGCB*, Chr4:52904225-52904560; *SGCA*, Chr17:48243242-48243570).

## Results

The NGS analysis of the proband revealed 3 heterozygous variants (Supplementary Figure 1). Two variants were localized in *CAPN3*, namely NM_000070.2 (*CAPN3*): c.550delA (p.Thr184Argfs) and c.1813G>C (p.Val605Leu) in the exons 4 and 16, respectively. The first is a single nucleotide deletion and is the most common (75% of cases) pathogenic variant in European Countries (1). As expected, the bioinformatic analysis classified the c.550delA (rs80338800) as a loss-of-function variant (p.Thr184Argfs), causing a frameshift of the open reading frame. The predictive tools (Mutation Taster, HSF, Varsome, PolyPhen 2, SIFT) described the variant as deleterious. In addition, SMART revealed that the altered protein product lacks the calpain 3 domain and the three “EF-hands” motifs. The first domain is involved in the signaling pathways of Calpain while the EF-hands are essential for the Ca^++^-dependent activation of the protein (4).

Concerning c.1813G>C, it is a novel missense variant (p.Val605Leu) which has been predicted to be deleterious (by Mutation Taster, HSF, Varsome, PolyPhen 2, SIFT) because of a potential alteration of splicing. This variant is not annotated in literature or among online databases and it has not been found in 200 control subjects. Unfortunately, the analysis by SMART tool did not yield significant results, as the variant is located within an uncharacterized domain. The segregation analysis of *CAPN3* showed that the mother and the maternal uncle were both heterozygous for c.550delA, while the father was carrier of c.1813G>C.

In addition, NGS analysis on the proband revealed the presence of a novel variant in *LMNA*, namely NM_170707 (*LMNA*): c.550C>T (p.Gln184^*^). The over-mentioned variant has been predicted to be a null variant, has not yet been described in literature or among online databases and it has not been found in 200 control subjects. Predictive tools (Mutation Taster, HSF, Varsome, PolyPhen 2, SIFT) described a pathogenic effect of this variant on the protein product. SMART tool reported that the altered LMNA protein lacks the filament domain, which is essential to maintain its structure and function. The segregation analysis highlighted the presence of the c.550C>T variant only in the mother, suggesting a possible association with her cardiac symptomatology and her family history of disease.

According to the criteria established by the American College of Medical Genetics (ACMG) Standards and Guidelines (23), c.1813G>C (*CAPN3*) can be described as a likely pathogenic variant considering that it is located in a critical domain without benign variation (PM1); it is absent in ExAc, GnomAD, and 1000 Genome Browser databases (PM2); considering recessive model of inheritance, it has been detected in trans with a pathogenic variant (PM3); multiple lines of computational evidence support a deleterious effect on the gene or gene product (PP3). Concerning the clinical classification of c.550C>T (*LMNA*) by ACMG, it can be designated as a pathogenic variant, since it is a null variant causing loss of function (p.Gln184^*^) in *LMNA* (PVS1); it is absent in ExAc, GnomAD, and 1000 Genome Browser databases (PM2); it is located in a critical domain without benign variation (PM1); multiple lines of computational evidence support a deleterious effect on the gene or gene product (PP3).

## Conclusions

This case report presented a patient affected by LGMD, who was found to be carrier of mutations not only in *CAPN3* (c.550delA and c.1813G>C) but also in *LMNA* (c.550C>T). These results explain the neuromuscular phenotype of the proband because of *CAPN3* mutations and highlight a potential risk of cardiovascular disorders due to the presence of the variant in *LMNA* and her positive family history. Given these results, the family members were subjected to the segregation analysis. In particular, the mother resulted to be carrier of *CAPN3*_c.550delA and *LMNA*_c.550C>T, respectively. The mother can be considered as a healthy carrier for the *CAPN3*_c.550delA pathogenic mutation associated with calpainopathy. However, the presence of a novel variant in *LMNA* may explain her cardiovascular pathology (bradycardia and syncopal episodes). The absence of neuromuscular symptomatology in the mother and the peculiar clinical picture of the proband excluded a possible association of *LMNA*_c.550C>T with neuromuscular phenotype, especially concerning Emery-Dreifuss Muscular Dystrophy (EMD). In fact, EMD specifically affects vastus lateralis and biceps brachii muscles which are relatively spared in LGM2A (24, 25).

**Figure 3:**
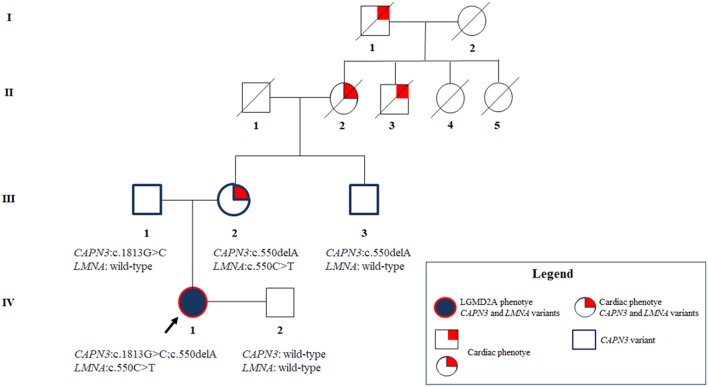
Pedigree showing the positive familiarity for cardiac phenotype inherited by maternal lineage and the transmission of the *CAPN3* and *LMNA* variants throughout the family members.

The maternal uncle was heterozygous only for the *CAPN3_*c.550delA, and, as expected, did not show any neuromuscular or cardiovascular problems. The father resulted to carry the novel *CAPN3_*c.1813G>C and, thereby, he was unaffected. Overall, segregation analysis confirmed the inheritance of the three mutations of the proband from her relatives and highlighted a familiarity for cardiomyopathy which cannot be neglected (Figure 3).

Altogether, these data raise some important considerations. First, NGS panel in this patient was critical to reach the molecular diagnosis of calpainopathy, detecting one known mutation and a second novel variant predicted as pathogenic. Second, NGS panel allowed the identification of the novel *LMNA*_c.550C>T variant in the proband. This result was consistent with the positive family history and with segregation data, explaining cardiovascular disease in the mother and, more importantly, recommending more specific cardiological follow-up in the proband. It is important to note that cardiological manifestations occurred in the mother later in age (55 years), so that we can suppose that the proband could still be in an “asymptomatic cardiological phase.” However, a variable phenotypic expression of *LMNA* mutation in the proband compared to the mother can't be excluded. All these data underline the importance of an integrated approach between clinicians and geneticists, for correct interpretation of results, proper genetic counseling and, eventually, clinical management and follow-up. With regards to genetic and familial counseling, the NGS analysis was also performed on the partner of the proband, in order to estimate the reproductive risk for the couple. The partner was negative to all the 18 tested genes, meaning that he has 1/650 residual risk to be a healthy carrier for LGMD causative mutations, considering that the test is 84% sensitive. Taking into consideration the genetic profile of the proband and the sensitivity of the NGS test, the residual risk for the couple to have a child affected with calpainopathy is 1/1300. On the other hand, the *LMNA* pathogenic variants are transmitted according an autosomal dominant pattern, meaning that the proband has 50% probability to have heterozygous children. However, the clinical picture of the offspring cannot be certainly predicted as the *LMNA*_c.550C>T is a novel variant and its functional impact on the phenotype is still unknown.

In conclusion, this case report highlights the clinical utility of NGS panels to provide accurate LGMD2A diagnosis and describe complex phenotypes and comorbidities originating from the inheritance of different mutations in multiple genes. However, the application of NGS in the clinical practice should always be combined with a pre- and post-genetic counseling in order to provide a clear explanation of the results, the possible implications on patients' phenotype, the recurrence risk within the family as well as to explain possible unexpected findings.

## Data Availability

No datasets were generated or analyzed for this study.

## Ethics Statement

This study was carried out in accordance with the recommendations of the ethics committee of Santa Lucia Foundation with written informed consent from all subjects. All subjects gave written informed consent in accordance with the Declaration of Helsinki. The protocol was approved by the ethics committee of Santa Lucia Foundation.

## Author Contributions

RC, CSt, VC, GC, RG, GPa, SC, CP, and JM made contributions to acquisition of data, analysis, and interpretation of data. CSt, RC, GC, RG, GM, and EG have been involved in drafting the manuscript. SZ, GPr, CSa, and SS have been involved in the acquisition of clinical data. RC, CSt, VC, GC, RG, GPa, SC, CP, JM, SZ, GPr, GM, CSa, SS, and EG have given final approval of the version to be published.

### Conflict of Interest Statement

The authors declare that the research was conducted in the absence of any commercial or financial relationships that could be construed as a potential conflict of interest.
