# Supplementary figures and images for: Limb-Girdle Muscular Dystrophies (LGMDs): The Clinical Application of NGS Analysis, a Family Case Report
Source: Front Neurol. 2019 Jun 13;10:619. doi: 10.3389/fneur.2019.00619 (PMC6585112; doi:10.3389/fneur.2019.00619)

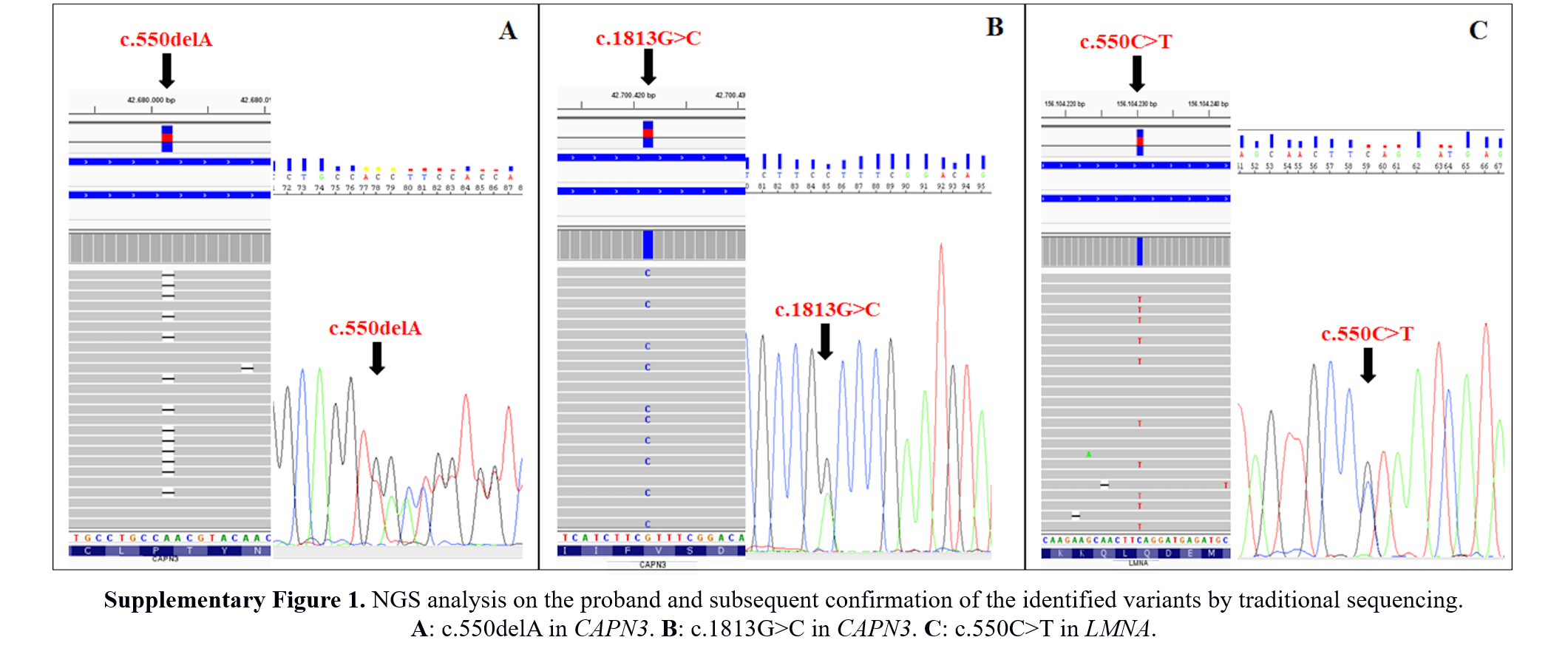

Supplement: Supplementary file 1 [file Image_1.TIF]
